# Isoaspartate formation and irreversible aggregation of collapsin response mediator protein 2: implications for the etiology of epilepsy and age-related cognitive decline

**DOI:** 10.21203/rs.3.rs-5074866/v1

**Published:** 2024-11-14

**Authors:** Jeff X. Zhu, Dana W. Aswad

**Affiliations:** University of California Irvine; University of California Irvine

**Keywords:** aging, autoimmunity – epilepsy, protein aggregation, protein damage, protein repair

## Abstract

Collapsin response mediator protein 2 (CRMP2) functions in the genesis and activity of neuronal connections in mammalian brain. We previously reported that a protein coincident with CRMP2 on 2D-gels undergoes marked accumulation of abnormal L-isoaspartyl sites in brain extracts of mice missing the repair enzyme, protein L-isoaspartyl methyltransferase (PIMT). To conflrm and explore the signiflcance of isoaspartyl damage in CRMP2, we expressed and purifled recombinant mouse CRMP2 (rCRMP2). A polyclonal antibody made against the recombinant protein precipitated CRMP2 from brain extracts of PIMT-KO mice, but not from WT mice, suggesting that (1) the rCRMP2 antigen underwent signiflcant isoAsp formation in the process of antibody production and (2) the isoAsp form of CRMP2 is considerably more immunogenic than the native protein. *In vitro* aging of rCRMP2 at pH 7.4, 37°C for 0–28 days led to robust accumulation of isoAsp sites that were repairable by PIMT, and also induced a progressive accumulation of apparent dimers and higher-mass oligomers as judged by SDS-PAGE. A similar pattern of CRMP2 aggregation was observed in mice, with levels increasing throughout the lifespan. We conclude that CRMP2 is indeed a major target of PIMT-mediated protein repair in the brain; that isoAsp forms of CRMP2 are highly immunogeni; and that CRMP2 dysfunction makes a signiflcant contribution to neuropathology in the PIMT-KO mouse.

## Introduction

The collapsin response mediator protein (CRMP) family is a group of phosphoproteins identifled primarily in the nervous system. The CRMP proteins are highly conserved and their expression is differentially regulated during neuronal development and maturation (Byk et al. 1998; Wang et al. 1996; Bretin et al. 2005; Veyrac et al. 2005). It is proposed that they play important and complementary roles in regulating neuron differentiation, axon outgrowth, and plasticity of the nervous system. Among all flve isoforms, the biological role of CRMP2 is most studied. Initially identifled as CRMP-62 in chick as the homologue of UNC-33 in *Caenorhabditis elegans* (Goshima et al. 1995), CRMP2 was also found and named as TOAD-64 in rat (Minturn et al. 1995, ULIP2 in mouse (Byk et al. 1996), and DRP-2 in human (Hamajima et al. 1996). This protein is strongly upregulated both developmentally and in NGF-induced PC12 cells, and is the major CRMP isoform present in post-mitotic neurons and glial cells of the adult brain (Kamata et al. 1998).

Increasing evidence suggests that CRMP2 plays a crucial role in neuronal differentiation, axon outgrowth, and guidance. CRMP2 mediates growth cone collapse induced by collapsin, lysophosphatidic acid (LPA) or ephrin-A5, which was shown to be mediated *via* phosphorylation sequentially by CDK5 and GSK-beta (Uchida et al. 2005) or by Rho-kinase (Arimura et al. 2000; Arimura et al. 2005) respectively. CRMP2 is also critical during neuron differentiation and axon outgrowth. Overexpression of CRMP2 led to strong neurite elongation in PC12 cells (Minturn et al. 1995), induced growth of multiple axons in cultured hippocampus neurons (Inagaki et al. 2001), and accelerated regeneration of injured motor neurons (Suzuki et al. 2003). More recent data indicates that the interaction of CRMP2 with cytoskeleton proteins plays an important role in dynamic cytoskeleton remodeling. CRMP2 facilitates the transport of tubulin from the cell body to the growing axon and promotes microtubule assembly by its interaction with heterotubulin dimers and cargo protein kinesin-1 (Fukata et al. 2002; Kimura et al. 2005). It was also involved in kinesin-1-dependent transport of Rac1-associated protein 1(Sra-1)/WAVE1 complex that modulated actin cytoskeleton (Kawano et al. 2005). Furthermore, CRMP2 was shown to interact with Numb, and regulate Numb-dependent endocytosis of neuronal cell adhesion molecule L1 during axon growth (Nishimura et al. 2003). The biological roles of CRMP2 in cytoskeleton dynamics and Numb-dependent endocytosis were shown to be negatively regulated by GSK3β-mediated phosphorylation in the PI3K/Akt/GSK3β signaling pathway (Nishimura et al. 2003; Yoshimura et al. 2005).

Aberrant expression and modiflcation of CRMP2 has been implicated in a number of neurological disorders. CRMP2 was found to be highly phosphorylated in the paired helical fllaments (PHF) of AD brain concomitant with elevated activities of Cdk5 and GSK3β (Gu et al. 2000; Cole et al. 2004). Reduced expression of CRMP2 was reported in the frontal cortex of patients suffering from schizophrenia, bipolar disorder, and depression (Johnston-Wilson et al. 2000). Conversely, upregulation of CRMP2 was observed in the rat hippocampus after treatment with anti-depression compounds venlafaxine or fluoxetine (Khawaja et al. 2004). In AD brain, CRMP2 was highly oxidized (Castegna et al. 2002). In the ischemic brain, wild type CRMP2 was cleaved at its C-terminus and the level of the modiflcation was associated with the duration of ischemic insult (Chung et al. 2005; Hou et al. 2006). Calpain was shown to cleave wild type CRMP2 in the NMDA-treated neuron cells (Bretin et al. 2006).

**Figure 1:**
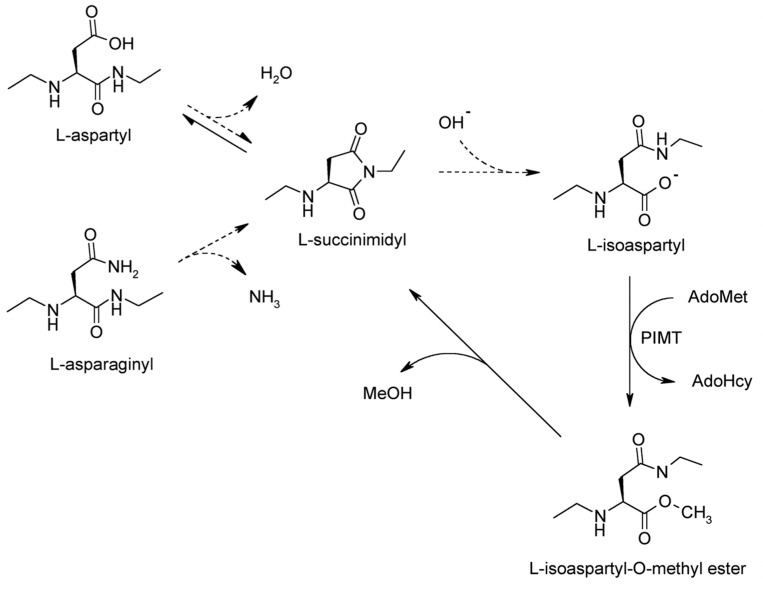
Mechanism of isoaspartate formation and PIMT-catalyzed repair. Under physiological conditions, deamidation of asparagine residues or dehydration of aspartic acid residues results in the formation of a metastable intermediate succinimide which spontaneously hydrolyzes to form a mixture of normal L-aspartyl and atypical L-isoaspartyl linkages. PIMT, using AdoMet as a methyl donor, selectively methylates the isoaspartyl α-carboxyl group to form a highly labile methyl ester. Spontaneous demethylation occurs within minutes to reform the original succinimide, with release of methanol as a by-product. This succinimide is now the starting point for further cycles of repair, resulting in near complete conversion of the isoaspartyl β-linkages to normal aspartyl α-linkages. Dashed lines indicate degradation steps, while solid lines indicate repair steps.

Formation of isoaspartate in proteins (Fig. 1) is a major form of spontaneous protein modiflcation and damage under physiological conditions (Aswad et al. 2000; Clarke 2003). Previously, we reported that CRMP2 appears to be a major endogenous protein highly susceptible to isoaspartyl formation as identifled in the PIMT-deflcient mouse brain (Zhu et al. 2006). Its biological signiflcance prompted us to further investigate the lability and biological consequence of isoaspartate formation. To facilitate the study, we cloned and expressed mouse rCRMP2, and performed *in vitro* aging studies on the purifled recombinant protein. Our flndings reveal a strong propensity of CRMP2 for isoaspartyl formation and aggregation both *in vitro* and in *vivo*, with implications for the etiology of epilepsy and age-related cognitive decline.

## Materials and Methods

### Materials

Frozen brains from PIMT-WT and PIMT-KO mice were provided by the laboratory of Dr. Mark Mamula at Yale University. Creation of the founder mice is described in Kim et al. (1997). S-[Methyl-^3^H]-Adenosyl-L-Methionine (AdoMet) and [γ−^32^P] ATP were purchased from Perkin Elmer (Boston, MA). ECL Anti-rabbit IgG, horseradish peroxidase-linked whole antibody, and ECL-Plus Western Blotting Detection System were bought from Amersham Biosciences Corp. (Piscataway, NJ). Immobilon-P PVDF membrane (0.45 um) was purchased from Millipore Inc. (Bedford, MA). Mouse brain cDNA library was from BD Clontech (Mountain View, CA). Recombinant human CDK5/p35 was purchased from Upstate Biotech (Lake Placid, NY). Sulfolink Coupling Gel was purchased from Pierce Biotech. Inc. (Rockford, IL).

### Cloning, expression, and purification of mouse PIMT

Full length cDNA coding sequence of CRMP2 was amplifled from mouse cDNA library (Clontech) using a forward primer containing an NheI site, and a reverse primer containing an XhoI site (the forward primer: 5’-GAGCTAGCATGTCTTATCAGGGGAAGAAAA-3’, and the reverse primer: 5’-GCCTCGAGTTTAGCCCAGGCTGGTGATG-3’). The amplifled PCR products were subcloned into pET28a(+) between NheI and XhoI after the restriction digestion. Plasmid containing murine CRMP2 was transformed into *E. coli* BL21(DE3) competent cells. The transformed cells were grown in the LB medium containing 50 μg/ml kanamycin at 37°C, and were induced with 0.5 mM IPTG at 20°C when the cell density reached 0.8 at 600 nm. Cells were harvested after induction for 6 h, and kept at −80°C.

6xHis-CRMP2 was affinity-purifled from Ni-NTA column from cell lysates using an imidazole gradient from 20 mM to 250 mM imidazole in the elution buffer containing 50 mM Tris-Cl, pH 7.5, 0.3 M NaCl, 5% glycerol, and 5 mM 2-mercaptoethanol. The purifled protein was dialyzed in the buffer containing 25 mM Tris-Cl, pH 7.5, 50 mM NaCl, 5% glycerol, and 3 mM DTT, and loaded onto a Q-Sepharose column. The protein was eluted with a linear gradient from 50 mM to 500 mM NaCl in the buffer containing 25 mM Tris-Cl, pH 7.5, 5% glycerol, and 3 mM DTT. Fractions containing pure 6xHis CRMP2 were pooled, concentrated, and kept at −80°C. To remove his-tags, 6xHis CRMP2 was treated by incubating with thrombin at a ratio of 1 mg protein per unit at 4°C overnight. The cleaved solution was dialyzed, concentrated, and kept at −80°C.

#### In vitro aging

Purifled rCRMP2 at 0.5 mg/ml was incubated in the buffer containing 25 mM Tris-Cl pH 7.4, 150 mM NaCl, 5% glycerol, 3 mM DTT, 0.05% NaN_3_ at 37°C. Samples were removed and frozen at −80°C at each time point.

### Methylation reaction and diffusion assay

A typical reaction (50 μl) contained 0.1 M MES pH 6.2, 100 μM AdoMet at 600 dpi/pmol, 2 μM recombinant PIMT, and 2 μM isoAsp-DSIP peptide or 20 μl CRMP2 protein sample. The diffusion assay is based on the method by Chelsky et al. (1984). Briefly, methylation reactions were carried for 30 min at 37 °C, and stopped by adding 50 μl of a solution consisting of 0.4M sodium borate (pH 10.4), 4% SDS, and 2% methanol. After mixing, 50 μl of the solution was spotted on a 3X-folded fllter paper in the cap of a glass vial containing 2.5 ml scintillation liquid. Vials were incubated at 40°C for 1 hr. Caps containing the fllter paper were replaced with new caps, and the vials were counted for 2 min. in LS 6500 multi-purpose scintillation counter (Beckman).

#### Quantitation of isoAsp-CRMP2 after SDS-PAGE.

CRMP2 samples were methylated in a reaction containing 0.1 M MES pH 6.2, 50 μM [^3^H}AdoMet at 10,000 dpi/pmol, 2 μM PIMT at 30°C for 10 min. The reaction was stopped by adding 4X SDS-PAGE sample buffer and heated at 50°C for 10 min. Electrophoresis was carried out in a 12% NuPAGE gel (Thermo-Fisher) at 100 volts in the cold room to minimize hydrolysis of the protein methyl esters. After staining/destaining with Coomassie blue, the gel was soaked in water for 20 min, then in 1 M sodium salicylate pH 5.2 for 20 min. The gel was then dried in Gel Dryer Vacuum System (Fisher Scientiflc), and exposed to an X-ray fllm at −80°C.

### Generation and purification of a polyclonal antibody to rCRMP2

Purifled recombinant rCRMP2 protein was used as an antigen to generate antibodies in rabbits by Bethyl Laboratories Inc. (Montgomery, Texas). The antiserum showed the antibody titer against rCRMP2 at more than 500,000 (OD = 1.0) via ELISA. Anti-rCRMP2 antibodies were then purifled in our laboratory from the antiserum using an affinity column prepared by coupling the purifled rCRMP2 to agarose beads *via* the Pierce Sulfo-link kit.

### Western blot

After transfer, PVDF membrane was flrst blocked with 5% nonfat milk in TBST for 30 min, then incubated with rCRMP2 speciflc antibodies (0.10–0.25 μg/ml) in 5% nonfat milk for 1 hr followed by incubation with the detection antibody (Anti-rabbit IgG HRP-linked) for 1 hr at room temperature. The membrane was washed three times in TBST following the antibody incubation. ECL-Plus Western Detection System was used to detect the signals.

### Protein assay

Protein concentration was determined using the Lowry protein assay (Waterborg and Matthews 1994) with BSA as the standard. Proteins were routinely precipitated with 7% (w/v) trichloroacetic acid prior to the assay to remove interfering substances.

## Results

### Expression of recombinant mouse CRMP2

**Figure 2:**
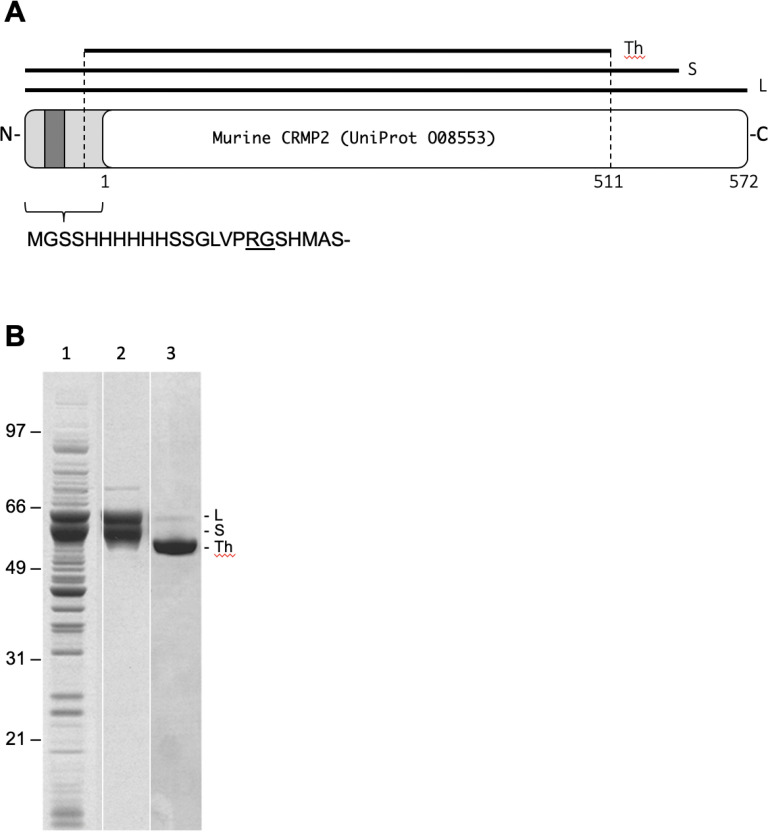
Expression and puriflcation of recombinant murine CRMP2 (rCRMP2). (A) Diagram of the 6xHis-CRMP2 construct showing the full length (L), short (S) and thrombin-trimmed (Th) forms. Key residue numbers for the native protein are shown along the bottom edge. (B) SDS-PAGE of samples before and after puriflcation; *E. coli* extract (Lane 1), purifled rCRMP2 (Lane 2), and purifled rCRMP2 after treatment with thrombin.

To determine if CRMP2 is susceptible to isoaspartyl formation *in vitro*, we cloned, expressed, and purifled recombinant mouse CRMP2 (rCRMP2) as described under Materials and methods. The full length cDNA of murine CRMP2 was cloned into pET28a (+) with a 6xHis tag at its N-terminus (Fig. 2A). The protein was expressed and purifled from *E. coli* BL21(DE3) cells containing the expression plasmid. We found that the expressed rCRMP2 showed two major bands between 60–64 kDa (Fig. 2B: lanes 1 and 2). The upper band is believed to be the entire CRMP protein (64 kDa) as expected, while the lower band appears to be a truncated form of the protein. The truncated CRMP2, seemed to be generated during expression and processing, was co-purifled with intact rCRMP2 after Ni-NTA and anion-exchange chromatography (Fig. 2, lane 2).

We assumed that the truncation form might be resulted from the proteolytic cleavage of a sequence highly susceptible to the protease. Coincidently, when thrombin was added to cleave the N-terminal 6xHis tags for the purifled proteins containing both the intact and truncated forms, we found that the cleavage resulted in a homogeneous species as shown by SDS-PAGE (Lane 3 in Fig. 1B). The homogeneous cleavage product corresponds to the thrombin-mediated cleavage at lysine 511 near the C-terminus (Fig. 1A) as determined by mass spectrometry. Taken together, these data indicate that proteolytic cleavage during expression occurred between Lys 511 and the carboxyl end.

Proteolytic lability has been reported by others for several members of the CRMP family. Recombinant CRMP1 was found to be cleaved at its C-terminus during expression in *E. coli* (Deo et al. 2004). Truncation of endogenous CRMP2 at its C-terminus was observed in NMDA-treated neurons (Hou et al. 2006), and in ischemic rat brain (Chung et al 2005), in which the cleavage at Lys 511 was observed for endogenous CRMP2. Although the biological role of the cleavage is unknown, the susceptibility of the C-terminus to the proteolytic cleavage suggests that the C-terminal domain might be involved in protein-protein interaction, and might be protected from endogenous proteolysis under normal physiological conditions *in vivo*.

### Immunoprecipitation of CRMP2 from PIMT WT and KO mouse brains

**Figure 3:**
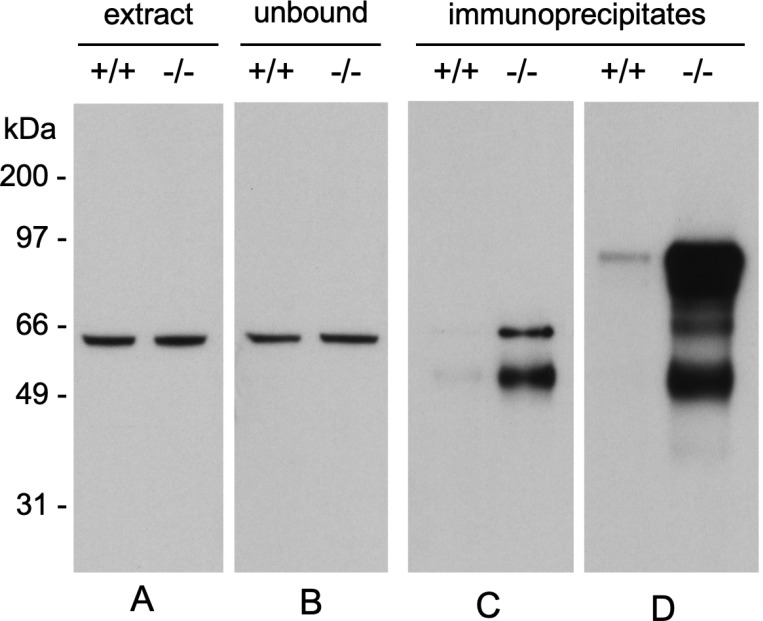
A polyclonal antibody made against rCRMP2 selectively precipitates CRMP2 from brain extracts of PIMT KO mice. (A) Equal amounts of CRMP2 are seen in a Western blot of brain extracts from WT and KO mice. (B) Equal amounts of CRMP2 are also seen in blots of the unbound material after immunoprecipitation. (C) Western blot of brain extracts immunoprecipitated with anti-rCRMP2 for 2 hours at 4 °C shows a marked selectivity of the antibody for the extracts from PIMT KO mice. (D) Western blot of proteins immunoprecipitated for 2 hours at room temperature, shows a marked selectivity of the antibody with the detection of apparent degradation and aggregation products. Preparation of the antibodies used here is described in Materials and methods.

The purifled rCRMP2 protein was used to as an antigen to generate polyclonal Abs as described in Materials and methods. Our goal was to assess isoAsp levels (*via* PIMT catalyzed ^3^H-methylation) in the immunoprecipitates (IPs) by immunoprecipitating CRMP2 from the brain extracts of both genotypes, in anticipation that the isoAsp content of from a PIMT KO mouse would be much higher than that from a PIMT WT mouse. Before attempting to measure isoAsp levels in the IPs, it was necessary to demonstrate that our pAb was equally effective in bringing-down CRMP2 from brain extracts of both genotypes. This was not the case. As shown in lanes C and D of Fig. 3, there was robust precipitation of CRMP2 from the KO extract, but almost nothing from the WT extract. The strong implications of this observation are that: (1) the purifled rCRMP2 we used as an immunogen underwent signiflcant isoAsp formation during the production process and immunization in the rabbit host; (2) the isoAsp form of CRMP2 is highly immunogenic, whereas the native form is a poor immunogen; (3) CRMP2 is a major endogenous substrate for the PIMT repair enzyme. Further evidence in support of these assumptions is presented below and in the Discussion.

#### rCRMP2 undergoes isoAsp formation and aggregation during in vitro aging

**Figure 4:**
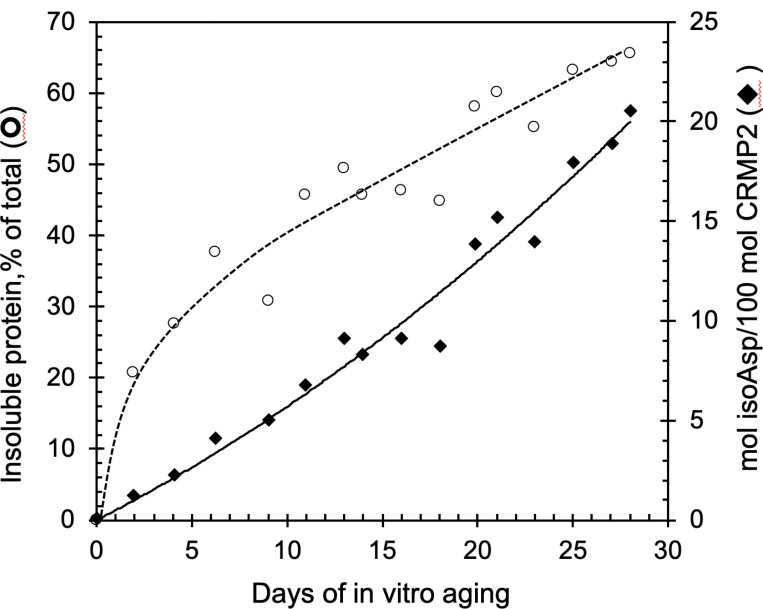
Formation of isoaspartates and loss of soluble rCRMP2 during *in vitro* aging. rCRMP2 was aged at pH 7.4, 37 °C for up to 28 days. The aged samples were centrifuged at 20,000 x g for 20 min. The collected supernatants were used to determine protein concentration and the determination of the isoaspartate content is described in Materials and methods.

Since isoaspartyl formation is a non-enzymatic reaction, *in vitro* aging under physiological conditions has been used as an effective model to mimic the isoaspartyl formation *in vivo* (Paranandi et al. 1994; Najbauer et al. 1996; Dimitrijevic et al. 2014). We subjected the purifled rCRMP2 to *in vitro* aging at 37°C, pH 7.4 for up to 28 days. The samples were then subjected to the centrifugation at 20,000 x g for 20 min, and the supernatants were assessed for protein concentration and isoAsp content. We found that the isoAsp content in soluble forms of rCRMP2 was increased in a near-linear fashion during the 28-day incubation (Fig. 4). Using an isoAsp-DSIP peptide as a standard, we measured 20 mol of isoAsp sites per 100 mol of soluble rCRMP2 after 28 days of incubation.

**Figure 5:**
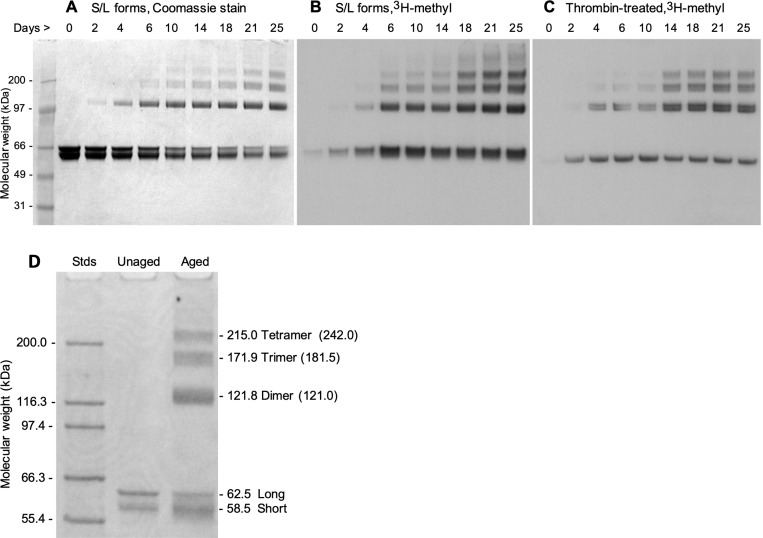
SDS-PAGE of rCRMP2 reveals progressive oligomer formation coincident with isoaspartate formation during *in vitro* aging. All lanes contained 3.3 μg proteins from the supernatants (as described in Fig. 4) of the aged rCRMP2 samples. (A) Coomassie blue staining of aged rCRMP2 containing the S and L forms. (B) Autoradiogram after SDS-PAGE of aged rCRMP2 after ^3^H-methylation with PIMT prior to electrophoresis to label the isoAsp sites. (C) Same as B, except the aged samples subjected to the thrombin treatment. (D) SDS-PAGE of unaged and aged (18 days) rCRMP2 using a different set of mass standards to more accurately estimate the molecular weights of the high mass bands seen in the aged samples. Mass numbers directly adjacent to the right of the gel were calculated from a 2nd-order polynomial flt (R^2^= 0.998) to a plot of log mol. wt. *vs* reference MWs for the flve standards shown. Numbers in parenthesis indicate the expected mass for oligomers based on the average mass (60.5 kDa) estimated for the monomeric S and L forms of CRMP2.

In addition, during the aging process, the protein underwent signiflcant structural change as shown in Fig. 4, in which the solubility of the protein continued to decrease (~ 65% were precipitated after 28 days), raising the possibility that the isoAsp formation might promote or facilitate aggregation. To further explore this idea, we aged rCRMP2 for up to 25 days, while taking periodic samples for analysis by SDS-PAGE (Fig. 5). The Coomassie stain in panels A and D show a progressive conversion of both the short and long forms of rCRMP2 to apparent dimers, trimers, and tetramers. Panels B and C reveal a concomitant increase in isoAsp levels in all the same oligomers of rCRMP2. This is consistent with, but by no mean proves, the possibility that the isoAsp formation plays a functional role in rCRMP2 aggregation. It is noteworthy these oligomers persist after the rCRMP2 samples are subjected to heating in SDS with 2-mercaptoethanol.

### PIMT repairs isoAsp sites in aged rCRMP

**Figure 6:**
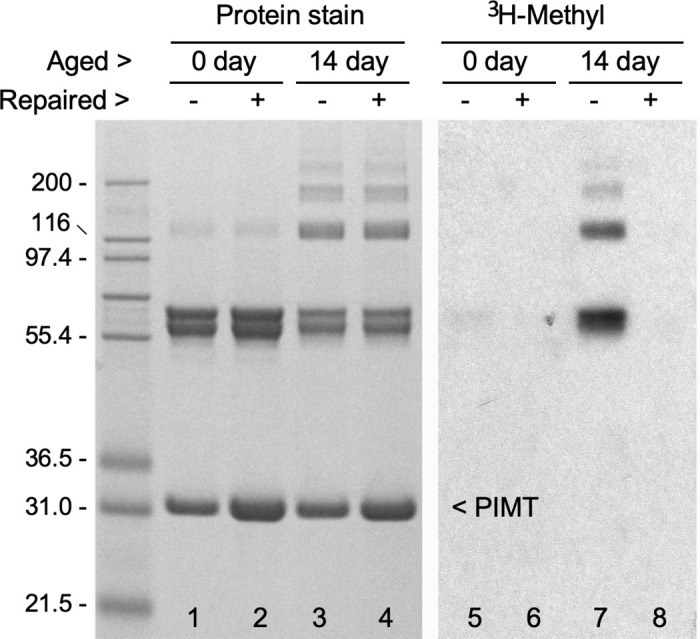
Demonstration that PIMT reverses isoaspartyl damage to rCRMP2 incurred by *in vitro* aging. Samples of control (0 day) and aged (14 day) rCRMP2 were incubated for 7 hours in a repair buffer (pH 7.8) with (+) or without (−) the addition of PIMT and unlabeled AdoMet. After the extensive dialysis to remove the AdoMet, equal amounts of fresh PIMT were added to all samples, along with [^3^H-methyl]AdoMet, and incubated in an assessment buffer (pH 6.2, needed to stabilize the ^3^H-methyl esters) to label any remaining isoAsp sites. Samples were then subjected to SDS-PAGE, followed by Coomassie staining (left panel) and fluorography (right panel). A comparison of lanes 7 and 8 indicates the presence of PIMT and AdoMet in the repair buffer resulted in nearly complete elimination of isoAsp in CRMP2 and its oligomers. Note that lanes 2 and 4 have 2X the amount of PIMT than in lanes 1 and 3, due to the addition of PIMT during the repair reaction. A more detailed description of the repair procedure can be found in Carter and Aswad (2008).

As further evidence that CRMP2 is an endogenous substrate for PIMT, we were able to demonstrate PIMT-catalyzed repair of isoAsp-damaged rCRMP2 *in vitro*. This is demonstrated in Fig. 6, using rCRMP2 that was aged for 14 days. Lane 4 shows the protein stain pattern of an aged sample that was subjected to a 6 h repair reaction (pH 7.4 and 37°C) in the presence of PIMT and unlabeled AdoMet. The control sample in lane 3 was subjected to a mock incubation in which no AdoMet or PIMT was added. After dialysis to remove unlabeled AdoMet from the repair sample, both samples were subjected to a second incubation containing [^3^H]-AdoMet and fresh PIMT to assess the presence of isoAsp sites (lanes 5–8). Lane 7 control shows the expected presence of isoAsp in the aged rCRMP2 control, while lane 8 shows a nearly complete absence of isoAsp in the repaired sample.

### Age-dependent oligomer accumulation of mouse brain CRMP2

**Figure 7:**
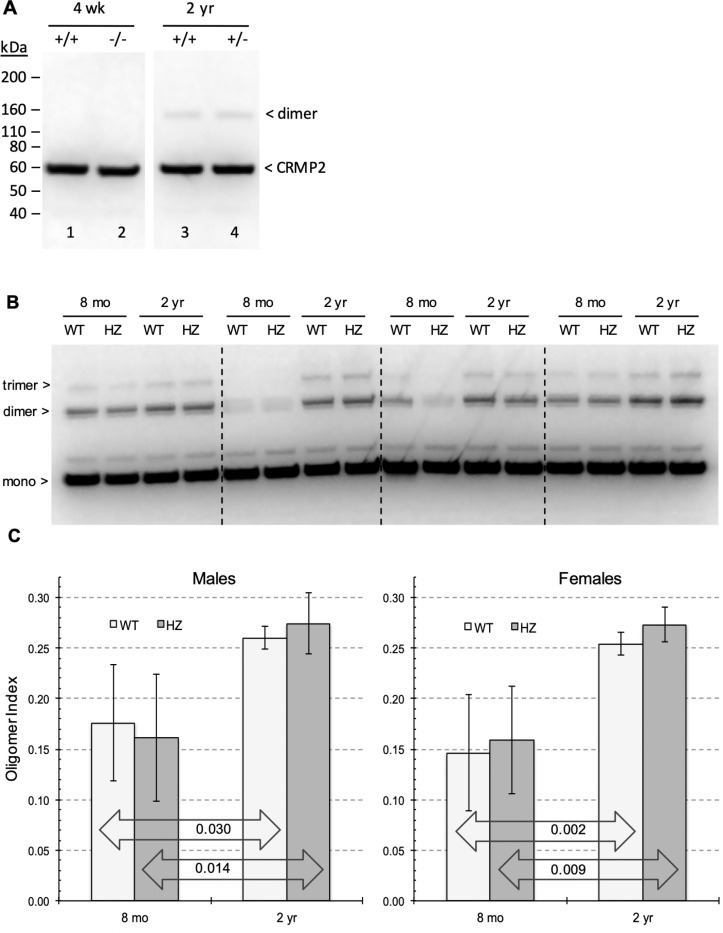
Comparison of CRMP2 oligomer formation in the mouse brain extracts. (A) Western blot for CRMP2 in brain extracts from PIMT +/+ and −/− mice, aged 4 weeks or 2 years. CRMP2 dimers are seen in the 2 years mice, and the amount of dimer is unaffected by loss of PIMT. (B) A comparison of CRMP2 oligomer patterns in PIMT +/+ vs +/− male mice at 8 months and 2 years. (C) A plot of oligomer index values [band density values of (dimer + trimer)/(monomer + dimer +trimer)] obtained from blots as shown in panel B.

Given the marked accumulation of rCRMP2 oligomers with *in vitro* aging, we wondered if (1) such oligomers are present in mouse brain, (2) if so, do they accumulate with age, and (3) does the level of PIMT activity affect oligomer formation. These questions are addressed in Fig. 7 for which we used our anti-rCRMP2 pAb to probe mouse brain extracts by Western blotting. Panel A (lanes 1 and 2) shows that CRMP2 oligomers were not detected at four weeks in either WT or KO mice. Four weeks was chosen because few KO mice survive any longer than that. At two years, dimers are seen at the same level in both WT and HZ mice; the latter in which PIMT levels are reduced by 45–50% (Qin et al. 2015). Panel B shows how oligomer content changes in mice between 8 months and 2 years. In this series, both dimers and trimers are seen here because the X-ray fllm was exposed longer than in panel A. Similar results were obtained when the panel B study was extended to female mice. Quantitation of these data (shown in panel C) indicates that (1) the oligomer formation was increased by 50–70% in mice between 8 months and 2 years in both genotypes, and in both sexes, and (2) the oligomer levels are considerably more varied in mice at 8 months than 2 years. The results from 2 year mice hint at a modestly higher increase in the oligomer levels in the HZ mice, leaving open the possibility that isoAsp formation does contribute to the oligomer formation.

## Discussion

We previously reported a proteomic analysis of PIMT substrates in the PIMT-KO mouse brain, suggesting that CRMP2 was among the three most abundant neuronal targets for the isoAsp repair enzyme PIMT (Zhu et al. 2006). Here we provide compelling new evidence that CRMP2 is indeed a major target of this enzyme, suggesting that isoaspartyl damage to CRMP2 contributes signiflcantly to the neuropathology and limited lifespan of the PIMT-KO mouse. This conclusion is based on our flnding that (1) purifled recombinant mouse CRMP2 is highly susceptible to isoaspartate formation when incubated at physiological pH and temperature, and (2) CRMP2 in the brain extract of PIMT-KO mice contain a damaged form that is highly immunogenic.

### Immunogenicity

**Figure 8:**
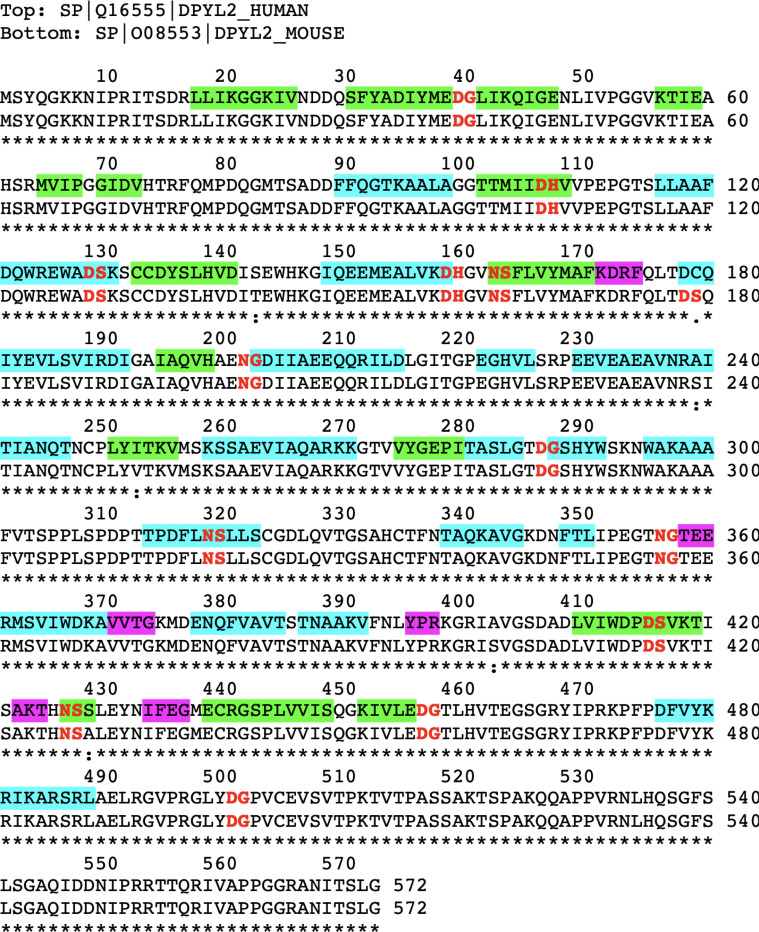
Sequence alignment of human and mouse CRMP2s, highlighted to show potential sites of isoAsp formation in relation to its secondary structure. Canonical hot spots for isoAsp formation are bold-faced in red. Regions of b-strands (green), a-helices (cyan), and turns (magenta), as seen in the Structural Features Viewer in UniProtKB for the mouse protein.

The high immunogenicity of CRMP2 in the KO mouse was gleaned from the IP results shown in Fig. 3. The antibody used here was made from recombinant CRMP2 raised against a rabbit host. We initially assumed that this antibody would immunoprecipitate both native and damaged forms of CRMP2 from mouse brain extracts, and that the immunoprecipitates from the KO mouse brain would show higher levels of isoaspartate than those from the WT mouse. In retrospect, this proved to be a naïve assumption, because the CRMP2 sequence is highly conserved among mammals, making it unlikely the rabbit would make a strong antibody response against the native form of CRMP2. Post-translational modiflcations are known to be associated with several autoimmune diseases (Doyle et al. 2007), and autoimmunity to histone H2B has been found in patients with lupus erythematosis, where it has been attributed to the presence of isomerization at the Asp^25^-Gly^26^ bond in the N-terminal tail (Doyle et al. 2013). As noted in Material and Methods, our rCRMP2 antigen generated a serum antibody response in rabbits with a titer of more than 500,000, making it a “super antigen”. It seems likely the multitude of isoAsp-prone sites in the sequence of CRMP2 (Fig. 8) is a contributing factor.

### Aggregation

During *in vitro* aging of CRMP2 we unexpectedly observed pronounced aggregation that temporarily correlated with the accumulation of isoaspartyl sites. The aggregation produced quantities of insoluble material in parallel with the formation of apparent dimers, trimers and tetramers as seen in Fig. 5.

Disulflde bond crosslinking is ruled out as the aging buffer contained 3 mM DTT, and the samples were heated for 10 min at 50°C in an SDS-PAGE sample buffer prior to electrophoresis. It is possible these aggregates arise from unfolding-induced, amyloid like structures, such as the β-aggregates observed by Majava et al. (2008) when human rCRMP2 was heated in the absence of divalent cations. An alternative is covalent crosslinking that does not involve disulfldes. An example of the latter would be crosslinks arising from the nucleophilic attack of a lysine sidechain (or N-terminal) amino group onto the succinimide intermediate that precedes isoAsp formation (Fig. 1). Such crosslinking has been seen in model peptides (Dehart and Anderson 2012), lysozyme (Desfougères et al. 2011), insulin (Brange et al. 1992), and ocular lens proteins (Friedrich et al. 2018).

We wondered if CRMP2 oligomers accumulate in the aging mouse brain with a pattern similar to that seen during *in vitro* aging of rCRMP2; and if so, is the degree of the oligomer accumulation affected by the level of PIMT activity (Fig. 7). Dimers and trimers are seen to accumulate dramatically between 8 months and 2 years of age, suggesting that CRMP2 aggregation is one of the many factors that cause brain function to decline with age. There is a hint in panel C that the reduced PIMT activity in the HZ mice (45–50% of WT activity) slightly increases the extent of the oligomer formation in the brain extract from 2 years old, providing an additional support for the involvement of isoAsp formation.

### Neuropathology and Aging

Similarities between PIMT-KO and CRMP2-KO mice further support the idea that decreased CRMP2 function contributes to the PIMT-KO neurological deflcits. Table 1 compares the phenotype of CRMP2-KO mice reported by Zhang et al. (2016) with corresponding studies carried out by various researchers on the PIMT-KO mouse.

In short, these mice are remarkably similar with regard to (a) thigmotaxis (atypical persistent walking around the perimeter of a novel enclosure, signifying reduced habituation), (b) decreased learning in the Morris water maze test, (c) enhanced susceptibility to epileptic seizures, (d) abnormal histology in the hippocampus and (e) normal (CRMP2-KO) or supra-normal (PIMT-KO) performance in the accelerating rotarod coordination test. Overall, the CRMP2-KO phenotype is milder than the PIMT-KO phenotype. Susceptibility to seizures is much lower in the CRMP2-KO, and they live a normal life span. This difference in phenotypic severity is expected as numerous proteins (probably 50 or more) are signiflcantly affected in the PIMT-KO. It is noteworthy that lacosamide, a drug useful in the control of epilepsy, may exert its action, at least in part, through interaction with CRMP2 (Wilson and Khanna 2015). CRMP2 promotes neurite outgrowth by direct interaction with tubulin (Gu and Ihara 2000; Fukata et al. 2002), and both of these proteins were identifled as top contributors to isoAsp accumulation in the PIMT-KO mouse (Zhu et al. 2006).

Several studies suggest that integrity of the PIMT repair system has a signiflcant effect on overall vitality in human aging.

1. In a study with postmortem human brain samples, PIMT enzyme activity showed a highly positive correlation (r = 0.51; p < 0.05) with age at death over the range of 20–80 years old. (Johnson et al. 1991).
2. In a population study of related individuals, David et al. (1997) found the variance (standard deviation/mean) of PIMT speciflc activity in human blood samples is extremely low (7.7%), compared with two other blood enzymes; histamine N-methyltransferase (24.1%) and thiopurine methyltransferase (21.6%). The PIMT variance is partly due to a common Ile/Val polymorphism at position 119, with the Val isoform showing slightly higher speciflc activity, and the Ile form showing greater thermal stability. The minimal variance of PIMT activity implies that low levels of PIMT are deleterious and possibly fatal in humans.
3. In a study of Askenazi jews, DeVry and Clarke (1999) compared the genotype frequency at PIMT position 119 in a relatively younger cohort (22–74 year), with that of an older cohort (75–104 year) that was aging well. The overall allele frequency was nearly identical between the two groups, but heterozygosity in the older cohort was higher (65%) than the 50% predicted by Hardy-Weinberg equilibrium. This was interpreted to mean that the heterozygotes had the best combination of PIMT speciflc activity, substrate speciflcity, and thermal stability.

In summary, the flndings reported here conflrm the identity of CRMP2 as a key target for PIMT-dependent protein repair in the brain, suggesting that its susceptibility to isoAsp damage contributes signiflcantly to the extreme neuropathology of the PIMT-KO mouse, and to age-related changes in overall health and cognitive function in humans.

## Figures and Tables

**Table 1:** 

Characteristic	PIMT-KO	CRMP2-KO
Open-fleld behavior	“hyperactivity in the open-fleld test” and “a strong thigmotaxic movement pattern” (Vitali and Clarke, 2004)	“showed increased locomotion” and “hyperactive in their home cages” (Zhang et al., 2016)
Coordination: accelerating rotorod	“perform signiflcantly better than their heterozygous and wild type litter-mates” (Vitali and Clarke, 2004)	“Motor coordination, balance and motor learning skills appeared to be intact” (Zhang et al., 2016)
Learning: Morris water maze	“impaired spatial memory” (Ikegaya et al., 2001)	“took a longer time to reach the hidden platform” (Zhang et al., 2016)
Hippocampal physiology: LTP	“mossy flber-CA3 synapses failed to show long-term potentiation or paired-pulse facilitation” (Ikegaya et al., 2001)	“Theta-burst stimulation (TBS)-induced longterm potentiation (LTP) was substantially reduced” (Zhang et al., 2016)

## Data Availability

The datasets used and/or analysed during the current study are available from the corresponding author upon reasonable request.
